# Functional Outcomes After Injections of Platelet-Rich Plasma for Plantar Fasciitis

**DOI:** 10.7759/cureus.83276

**Published:** 2025-04-30

**Authors:** Harpreet Singh, Sangam Tyagi, Rayed Qamar, Mit Parikh, Akshit Sen, Kavya Joshi, Ammar Rampurwala, Purvesh Bhrambhatt

**Affiliations:** 1 Orthopaedics, Geetanjali Medical College and Hospital, Udaipur, IND; 2 Orthopaedics and Traumatology, Geetanjali Medical College and Hospital, Udaipur, IND; 3 Orthopaedic Surgery, Geetanjali Medical College and Hospital, Udaipur, IND; 4 Radiodiagnosis, Geetanjali Medical College and Hospital, Udaipur, IND; 5 Orthopaedics, Gujarat Medical Education and Research Society (GMERS) Medical College, Vadodara, IND; 6 Orthopaedics, Sawai Man Singh (SMS) Medical College, Jaipur, IND

**Keywords:** aofas, growth factor, healing, plantar fasciitis, platelet rich plasma, vas

## Abstract

Objective

Plantar fasciitis is a rather common disorder with symptoms like heel discomfort. It usually gets worse with prolonged rest. Platelet-rich plasma (PRP) injections, derived from autologous blood, have emerged as a promising treatment option by promoting tissue healing and reducing inflammation. This study evaluates the outcome of PRP injections in decreasing pain and improving patients’ function.

Methods

This study included 100 identified cases of plantar fasciitis altogether. After PRP injections, their results were evaluated using the American Orthopedic Foot and Ankle Score (AOFAS) at baseline and the visual analog scale (VAS) both at baseline and four, eight, and 12 weeks following injection.

Results

The mean VAS decreased to one from seven in males and from seven to one in females. A statistically significant difference existed between the males and females at four weeks (p ≤ 0.0001), eight weeks (p ≤ 0.0001), and 12 weeks (p = 1.0), and the AOFAS score reached 95/96 from 56/58 in males and females.

Conclusion

The efficacy of PRP in the treatment of plantar fasciitis is very high. It provides very good outcomes with significant pain relief while also promoting healing of the plantar fascia.

## Introduction

Plantar fasciitis (PF) is the primary cause of heel pain, affecting about two million people annually. The condition occurs when the strong tissue ring supporting the foot's arch becomes inflamed [1]. The plantar fascia is a connective tissue band that stretches from the calcaneus to the toes on the foot's inferior surface. It offers support for the medial arch, enhances shock absorption, and aids in walking coordination through the windlass mechanism [2]. PF, a condition impacting the plantar fascia, is often the source of heel pain. PF impacts not only athletes but also sedentary middle-aged individuals; nonetheless, the predominant risk factors include aging, obesity, excessive weight-bearing, and tight Achilles tendons [3]. Conservative therapies constitute the primary therapeutic approach, encompassing non-steroidal anti-inflammatory drugs, physiotherapy involving plantar fascia stretching exercises, activity modification, the application of shoe insoles, corticosteroid injections, and extracorporeal shock wave therapy [4,5]. Direct steroid injections into the plantar fascia represent a recognized and rapid therapeutic option [5]. New treatment plans should be viewed as more effective therapy alternatives since they promote a healing response rather than suppressing the inflammatory process. Platelet-rich plasma (PRP), which is widely known to promote cell development and subsequently tissue healing, has been used because of this. The goal of employing PRP is to boost the tendon's capacity for regeneration. It contains high concentrations of cells and cytokines in doses that are above physiological levels, which should encourage cellular chemotaxis, matrix synthesis, and proliferation [6]. Platelet concentration is mechanically increased by seven to 25 times over baseline values in whole blood thanks to centrifugation. These have prompted the use of PRP as a vector to transport growth factors to localized muscle and tendon injury and repair zones in an effort to promote and speed healing. The composition of PRP can vary based on the preparation method, but it generally includes the following components [7]. (1) Platelets: PRP often contains a greater concentration of platelets than standard blood. Normal blood platelet concentration is around 150,000 to 450,000 platelets per microliter, whereas PRP can have two to five times this concentration. (2) Growth factors: Platelets release growth factors and cytokines that facilitate tissue repair and regeneration. The key growth factors include platelet-derived growth factor (PDGF), transforming growth factor beta (TGF-β), vascular endothelial growth factor (VEGF), epidermal growth factor (EGF), and insulin-like growth factor (IGF). (2) Plasma: The liquid component of blood that carries cells and proteins throughout the body. It contains various proteins, nutrients, electrolytes, hormones, and waste products. Fibrinogen: (3) A protein essential for clot formation that serves as a scaffold for cell migration and tissue repair. (4) White blood cells (WBCs) (variable): Certain PRP formulations include leukocytes (leukocyte-rich PRP), potentially enhancing immune response and modulating inflammation. Some are deficient in leukocytes to reduce inflammation, contingent upon the particular use.

## Materials and methods

This prospective study was conducted at the Department of Orthopaedics, Geetanjali Medical College and Hospital (GMCH), Udaipur, following approval by the Institutional Ethics Committee. The study complied with the ethical principles specified in Schedule Y and the New Drugs and Clinical Trial Act of 2020, assuring adherence to clinical research standards. Participants were thoroughly apprised of the study's nature and aim in their preferred language, and written consent was secured. Confidentiality was maintained throughout the study. A total of 100 patients, regardless of gender, were included after failing twelve weeks of conservative treatment for PF, which included rest, physical therapy, and NSAIDs. Exclusion criteria included patients with systemic disorders, such as rheumatoid arthritis, malignant cancer, hematological disease, infection, or immune deficiencies, and recent administration of anticancer drugs or immunosuppressive drugs. The criteria also included a pain score of less than seven, an infection or ulcer at the injection site, and pregnancy. Patients with comorbidities like diabetes mellitus or severe cataracts for corticosteroid treatment were also excluded.

Preparation of PRP

**Figure 1:**
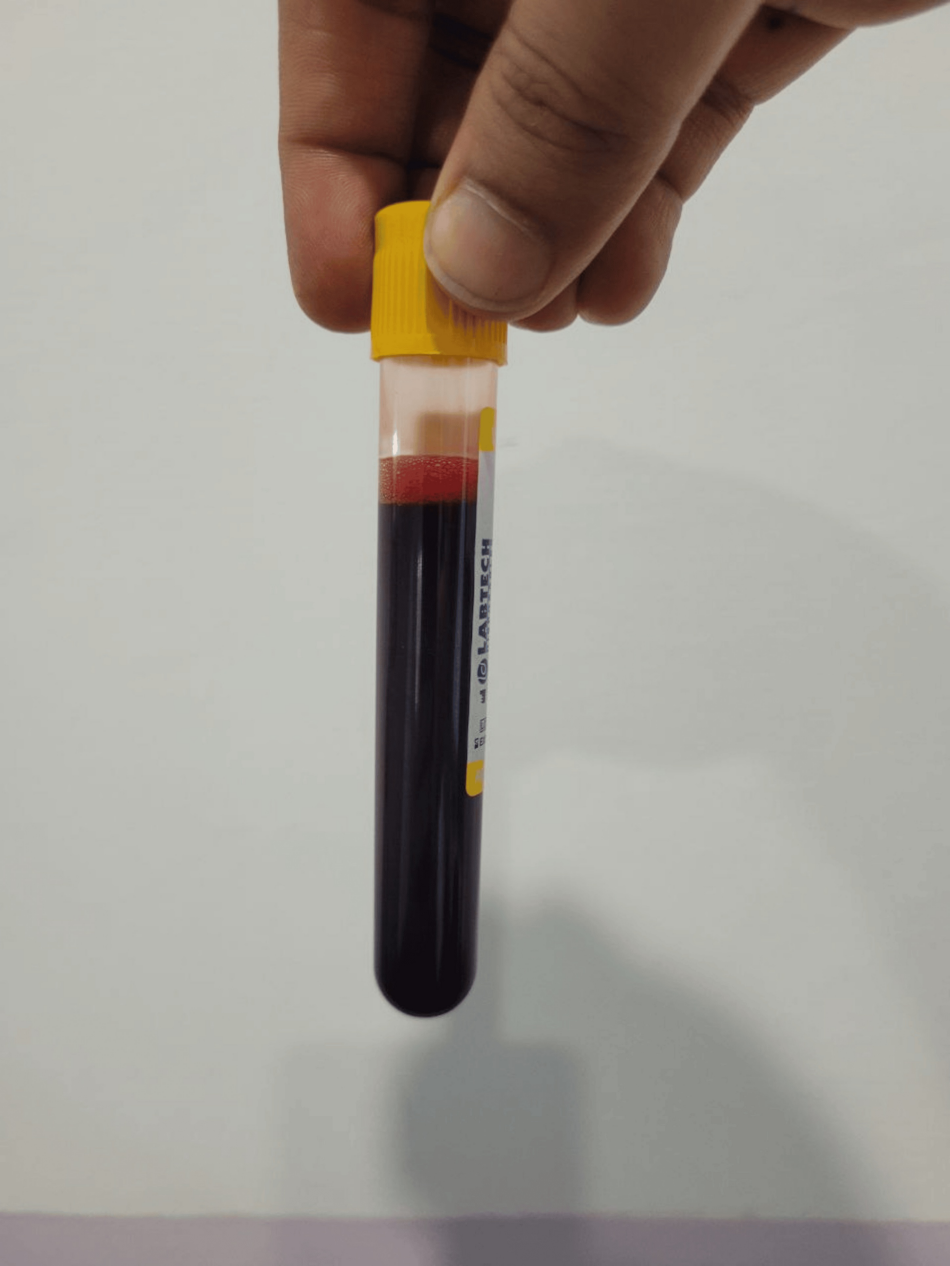
Blood-filled vacutainers

**Figure 2:**
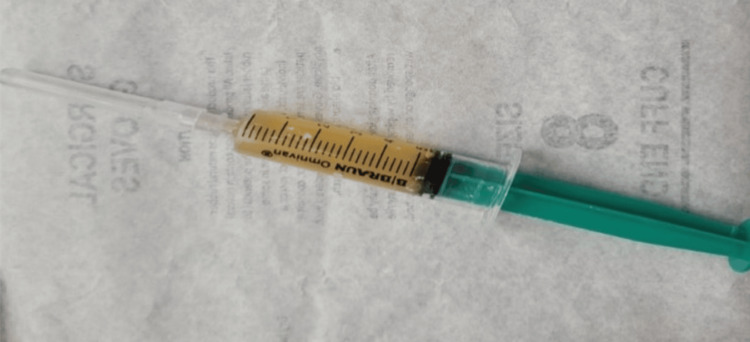
Platelet-rich plasma

PRP was produced utilizing the double spin centrifugation method described by Mazzocca et al [8]. The cubital vein was accessed, and 20 mL of blood was collected. The blood was promptly placed into vacutainers. The vacutainers were positioned in the centrifuge, ensuring proper counterbalancing. The preliminary centrifugation was conducted at 2400 revolutions per minute for 10 minutes, yielding a bifurcation of blood into two strata: a layer abundant in red blood cells at the bottom and a layer enriched with plasma and platelets at the top. The upper layer was meticulously transferred to new vacutainers with an 18-gauge needle and syringe. The vacutainers underwent a second centrifugation at 3600 revolutions per minute for 15 minutes, further isolating the plasma. This procedure produced a layer of platelet-poor plasma at the top and a layer of PRP at the bottom. Utilizing an 18G needle, the platelet-poor plasma from the superior portion was discarded, while the PRP from the inferior section was collected and readied for application (Figure 1 and Figure 2).

Method of injection

Preparation of the site: Clean the area with a povidone-iodine solution in a sterile manner.

Preparation of the injection: Using an 18-gauge needle connected to a 2-mL syringe, withdraw 2.5 mL of PRP. Switch to a 25-gauge needle.

Positioning the patient: Position the patient comfortably, ensuring the affected foot is stabilized. Locate the most sensitive area on the plantar fascia by gently palpating.

**Figure 3:**
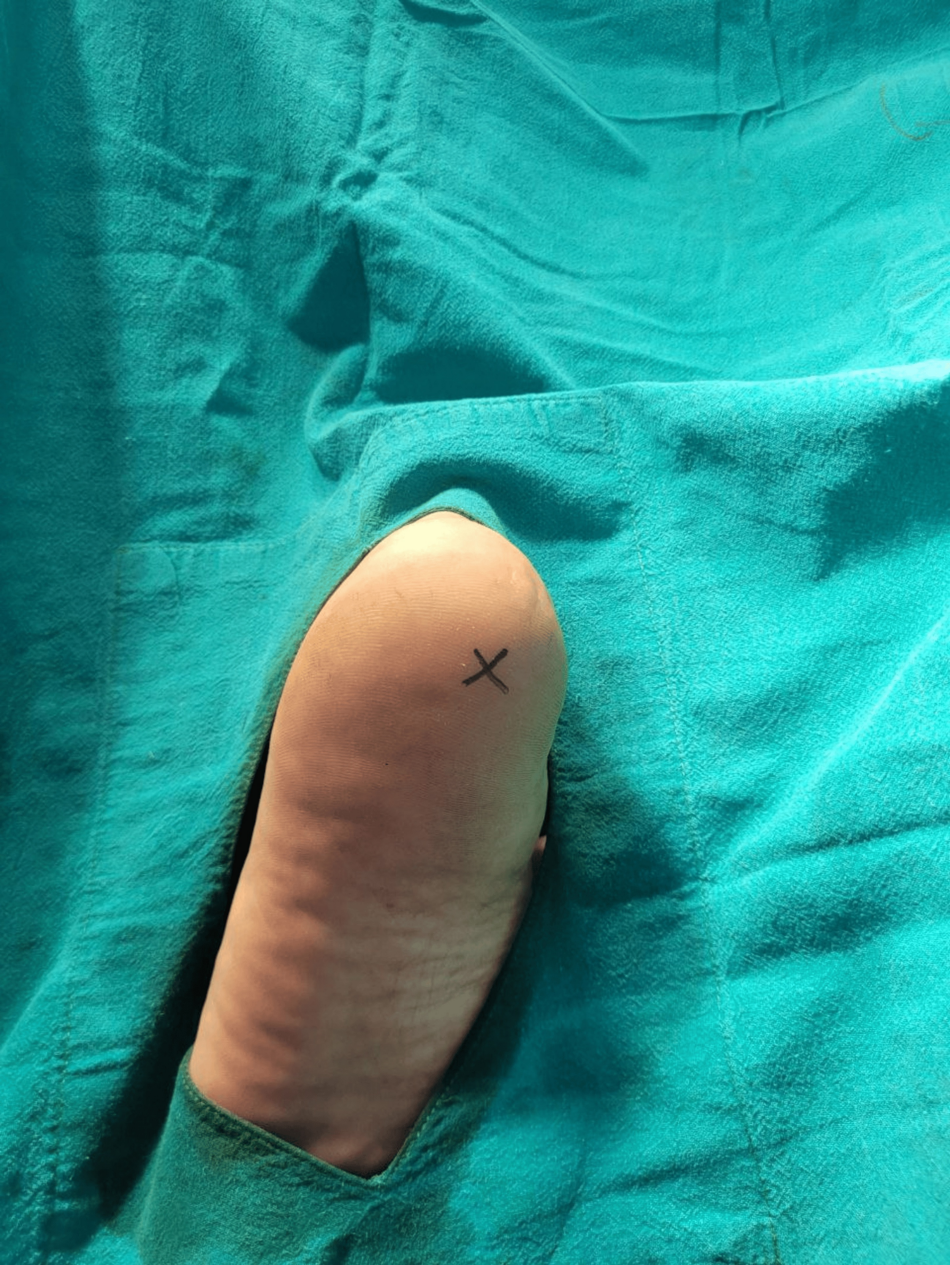
Preparation of injection site

Method of administering the injection: Insert the needle into the skin at a 90-degree angle, reaching the fascia. Aspirate to check for the presence of blood to ensure the needle is not in a vessel. If there is no blood, proceed with the procedure. Apply the PRP at the specified location, then gradually remove the needle. An antiseptic dressing should be applied to the injection site (Figure 3).

## Results

**Figure 4:**
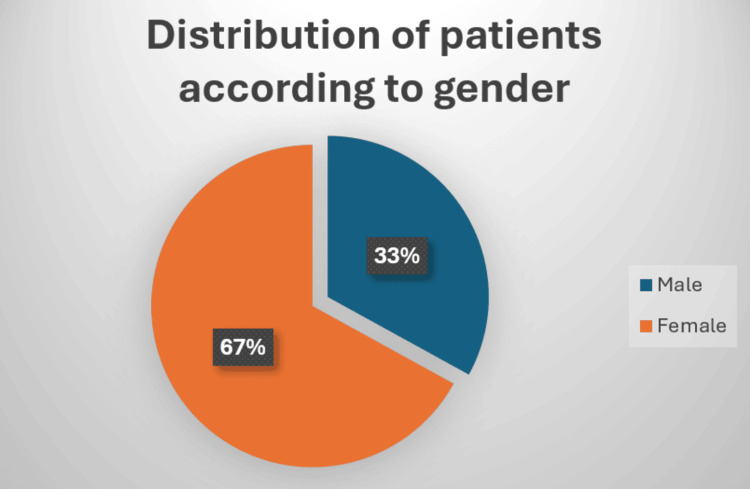
Gender distribution

The gender distribution indicates a higher prevalence of PF among females (Figure 4). There were 100 patients in total, of which 67 (67%) were female and 33 (33%) were male. 

**Figure 5:**
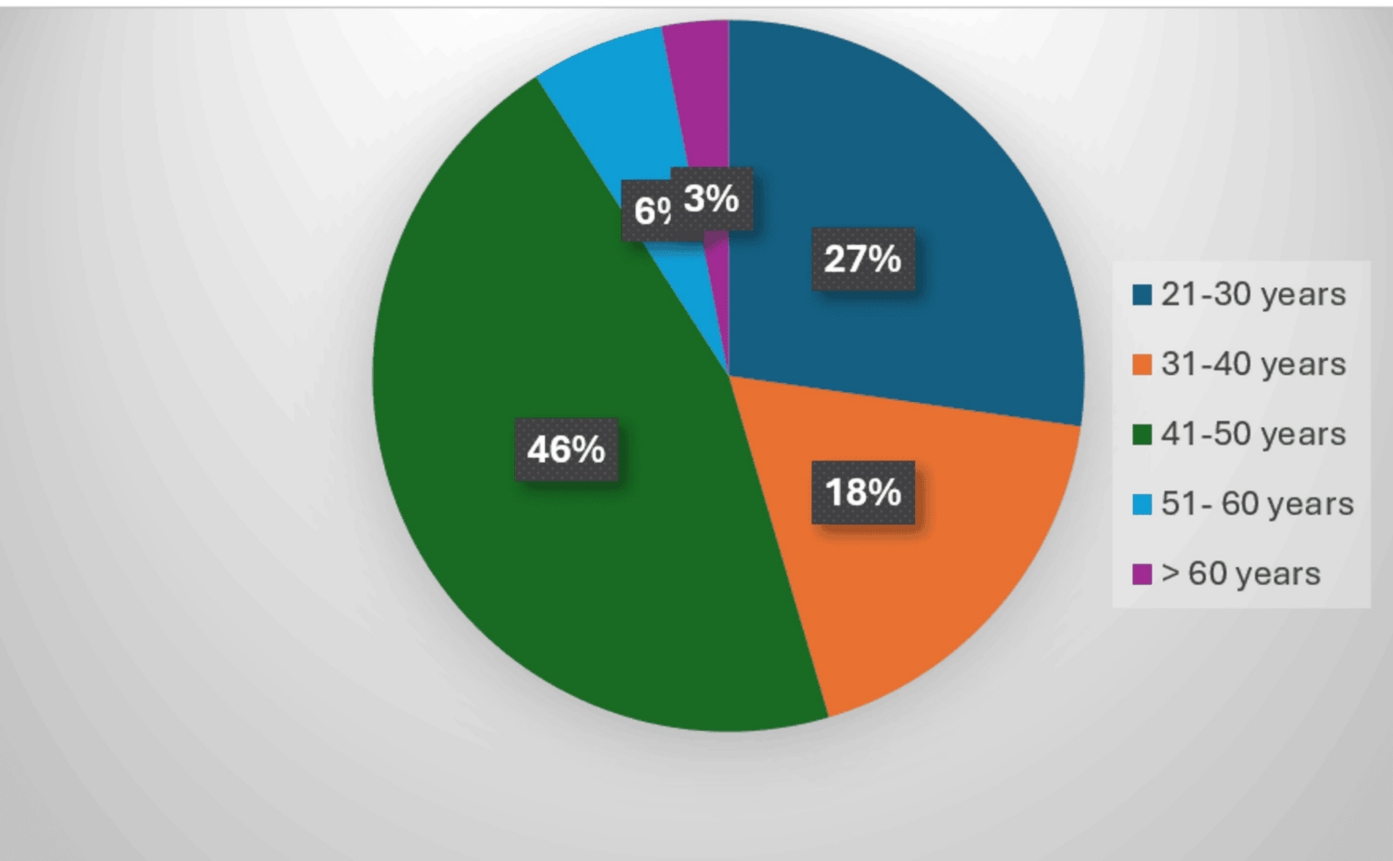
Distribution of patients according to age groups

There were 46 (46%) patients between 41 and 50 years, 27 (27%) were between 21 and 30 years, 18 (18%) were between 31 and 40 years, six (6%) were between 51 and 60 years, three (3%) were between more than 60 years of age (Figure 5).

The anthropometric parameters in male and female patients are presented in Table 1.

**Table 1:** Anthropometric parameters

S. no.	Obesity factor	Male (N = 33)		Female (N = 67)		P-value
		Mean	SD	Mean	SD	
1.	Height (m)	1.78	0.052	1.64	0.065	<0.0001
2.	Weight (kg)	92.60	5.1	77.40	2.50	<0.0001
3.	BMI (kg/m^2^)	29.09	1.82	28.57	1.24	0.096

The mean BMI of males was 29.09 kg/m^2^, and that of females was 28.57 kg/m^2^. The results in the above table indicate that both males and females with PF typically fall into the overweight category.

**Figure 6:**
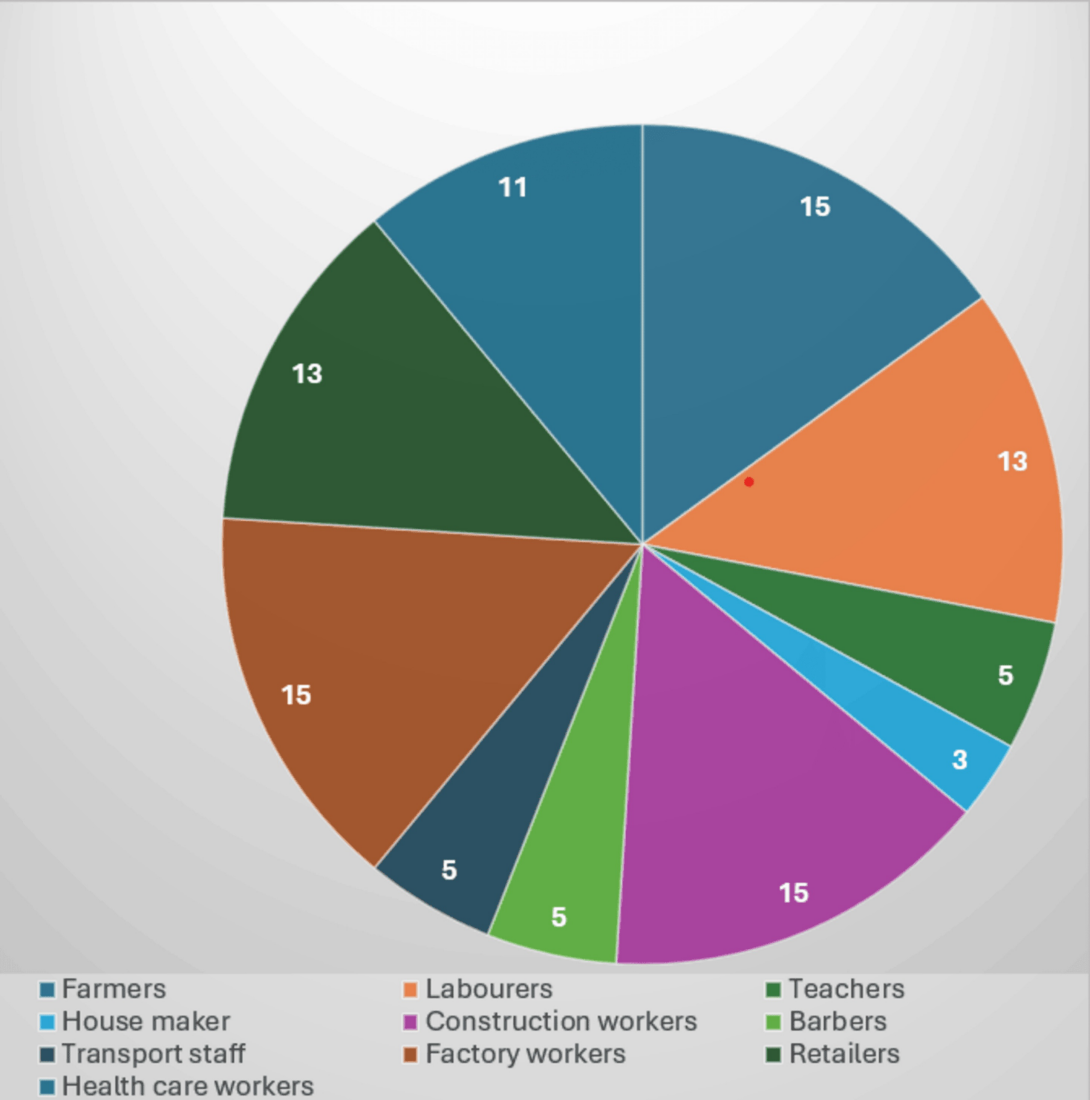
Distribution of patients according to occupation

In the study, the occupational distribution was as follows: healthcare workers - 14 females (14%) and 11 males (11%); retailers - 11 females (11%) and 13 males (13%); factory workers - 13 females (13%) and 15 males (15%); transport staff - two females (2%) and five males (5%); barbers - one female (1%) and five males (5%); construction workers - 11 females (11%) and 15 males (15%); homemakers - 16 females (16%) and three males (3%); teachers - 10 females (10%) and five males (5%); laborers - 10 females (10%) and 13 males (13%); and farmers - 12 females (12%) and 15 males (15%) (Figure 6).

At the beginning and in the first few weeks of treatment, the p-value analysis reveals that there are significant differences between the pain levels of males and females, with the former initially reporting more pain. Nonetheless, both genders saw comparable and noticeably lower pain levels by eight and 12 weeks, demonstrating the treatment's general efficacy for both genders (Table 2).

**Table 2:** The VAS score in platelet-rich plasma (PRP)-treated male and female patients *Since we are comparing means of two independent groups (males and females), a two-sample T-test is applied at a 0.05 significance level (p-value) and a 95% confidence level.

VAS score	Males (N = 33)		Females (N = 67)		P-value*
	Mean	SD	Mean	SD	
Pretreatment	7	0.90	8	1.02	<0.0001
0 week	6	0.90	7	1.60	<0.0001
4 weeks	5	0.65	4	1.10	<0.0001
8 weeks	3	0.95	2	0.86	<0.0001
12 weeks	1	0.79	1	0.66	1.0

Both genders' American Orthopedic Foot and Ankle Score (AOFAS) scores at 12 weeks are extremely high, indicating a very good recovery. It is interesting to note that at this stage, women have a slightly higher mean score than men. There is a statistically significant difference (Table 3).

**Table 3:** The American Orthopedic Foot and Ankle Score (AOFAS) score in platelet-rich plasma (PRP)-treated male and female patients *Since we are comparing means of two independent groups (males and females), a two-sample T-test is applied at a 0.05 significance level (p-value) and a 95% confidence level.

AOFAS score	Males (N = 33)		Females (N = 67)		P-value*
	Mean	SD	Mean	SD	
Pretreatment	58	2.16	56	1.87	<0.0001
0 weeks	65	0.92	64	0.95	<0.0001
4 weeks	89	1.21	88	1.05	<0.0001
8 weeks	95	1.39	94	1.67	0.0038
12 weeks	95	1.40	96	1.38	0.0010

## Discussion

The study was performed at the Department of Orthopedics, Geetanjali Medical College and Hospital (GMCH), Udaipur, to assess the effectiveness of PRP injections in treating chronic PF. The investigation comprised a total of 100 incidents. Distribution of patients on the basis of general characteristics like age, sex, occupation, involvement of side, and visual analog scale (VAS) and AOFAS score was noted, with the follow-up on the cases lasting from the day of treatment until 12 months; results are discussed here. The study focuses on the functional outcome of PRP injections in PF patients, emphasizing pain alleviation, functional improvement, and overall patient satisfaction.

Findings of gender-wise distribution of patients in different studies 

Our study shows a greater prevalence of females than males. Similar results were seen in Riddle and Schappert [9], Scher et al. [10], Hill et al. [11], Singh et al. [12], Sami et al. [13], Soraganavi et al. [14], and Naik et al. 2021 [15].

Findings of the mean age of patients in different studies 

Our study shows a mean age between 35 and 45 years in both genders. Similar results were found in Riddle and Schappert [9], Buchbinder [16], Hill et al. [11], Crawford et al. [17], Soraganvi et al. [14], Naik et al. [15], Kothari et al. [18], and Singh and Ummat [12].

Findings on the BMI of patients in different studies 

Our study shows that the average BMI value of affected females was 28.57 kg/m², while that of males was 29.09 kg/m² (Table 4).

**Table 4:** Findings of BMI of patients in different studies

S. no.	BMI values	References
1	28.1 kg/m^2^ in females and 29.3 kg/m^2^ in males	Prichasuk [19]
2	27.5 in females and 27.9 in males	Rome et al. [20]
3	27.8 kg/m^2 ^in females and 29.6 kg/m^2^ in males	Riddle and Schappert [9]
4	29.4 kg/m^2^ in females and 30.1 kg/m^2^ in males	Ozdemir et al. [21]
5	30.2 kg/m^2 ^in females and 28.5 kg/m^2^ in males	Hill et al. [11]

Findings of the occupation factor of patients in different studies

The findings of the occupation factor of patients in different studies are presented in Table 5.

**Table 5:** Findings of the occupation factor of patients in different studies

S. no.	Occupation factor	References
1	Teaching and health care	Hill et al. [11]
2	Teachers, factory workers, and health care providers.	Riddle and Schappert [9]
3	Farmers (15%) and homemakers (16%), respectively. Laborers (13% males and 10% females), factory workers (15% males and 13% females), teachers (5% males and 10% females), healthcare providers (13% males and 11% females), and construction workers (15% males and 11% females).	Our study

Findings of VAS score improvement in patients in different studies 

Our study shows improvement in the VAS score after 12 weeks. Similar improvements were also observed in the studies by Hurley et al., Acosta-Olivo et al., Jain et al., Omar et al., Sherpy et al., Tiwari and Bhargava, Uğurlar et al., and another by Jain et al. [22-29], as well as in studies by Kothari et al. [18], Singh and Ummat [12], Mahindra et al., Vahdatpour et al., and Say et al. [30-32].

Findings of AOFAS score improvement in patients in different studies 

Our study shows that the AOFAS score reached 95/96 from 56/58 in males and females. Similarly, improvement in scores was observed in the studies by Jain et al. [29], Mahindra et al. [30], Acosta-Olivo et al. [23], Say et al. [32], Ling and Wang [33], and Monto [34,35].

PDGFs and cytokines are essential in the healing process by augmenting collagen production, boosting bone cell proliferation, and facilitating fibroblast chemotaxis and activity. They stimulate macrophages, promote angiogenesis, and facilitate the chemotaxis of immune cells. Furthermore, they impede osteoclastogenesis and bone resorption, promote endothelial cell migration and mitosis, and enhance vascular permeability. These factors also facilitate the chemotaxis of macrophages and neutrophils, promote cellular proliferation, and enhance epithelial cell differentiation. Additionally, they stimulate cytokine release from mesenchymal and epithelial cells, facilitating cell proliferation, differentiation, and recruitment in diverse tissues such as bone, blood vessels, and skin. In combination with PDGF, they augment collagen synthesis, ultimately facilitating the regeneration and repair of the injured plantar fascia tissue. The limitations of this study include the small sample size and short-term results. A larger sample size and further investigation into the long-term effects of PRP are needed.

## Conclusions

The thorough examination of PF among different sexes reveals important demographic, anthropometric, and clinical findings. Women show a greater frequency, with a significant age range and slightly higher average age compared to men. Both sexes have similar biomechanical risk factors, highlighting the importance of occupation and foot traits in the prevalence of diseases. Results from treatment, as indicated by VAS and AOFAS scores, show good progress with significantly superior results noted in females. PRP takes more time to act, but the effect of PRP is more durable with time and is reliable. The findings suggest that PRP therapy is superior for treating PF, as it alleviates pain more effectively. The study lasted 18 months, and for enhanced validity, it necessitates a larger patient cohort and additional time. This research topic needs more research for better and more conclusive results. The study was single-centered, so external validity could not be guaranteed, and there was no control group.
